# I Have a Fear of Negative Evaluation, Get Me Out of Here! Examining Latent Constructs of Social Anxiety and Autistic Traits in Neurotypical and Autistic Young People

**DOI:** 10.1007/s10803-020-04657-3

**Published:** 2020-08-18

**Authors:** Jiedi Lei, Ailsa Russell

**Affiliations:** Department of Psychology, Centre for Applied Autism Research, University of Bath, Bath, BA2 7AY UK

**Keywords:** Autism spectrum disorder, Autistic disorder, Social phobia, Social anxiety disorder, Diagnostic self-evaluation

## Abstract

Understanding shared and unique constructs underlying social communication difficulties in autism spectrum disorder (ASD) and social anxiety disorder (SAD) can address potential diagnostic overshadowing when evaluating SAD in the context of autism. Using self-report measures, factor analyses examined constructs underlying autistic traits, social anxiety, internalising symptoms and wellbeing amongst 267 neurotypical (17–19 years) and 145 autistic (15–22 years) students in the UK. Shared constructs across measures assessed general social communication competency (e.g., social distress in new situations and peer relationships). Fear of Negative Evaluation (FNE) was identified in both samples as a stable construct unique to social anxiety. Adapting interventions targeting SAD in autism should target FNE during adolescence which marks a period of heightened peer interaction and social vulnerability.

Autism spectrum disorder (ASD) is characterised by social and communication difficulties and restricted and repetitive behaviours and interests (American Psychiatric Association 2013). Between 29.2 and 57% of autistic^1^ children and adolescents experience clinically significant level of social anxiety disorder (SAD) (Bellini 2004; Kuusikko et al. 2008; Simonoff et al. 2008), a prevalence rate that is considerably higher than the 7.1% to 12.1% reported in the neurotypical population (Izgiç et al. 2004; Ruscio et al. 2008). Social anxiety is thought to exacerbate the effects of social impairment and social functioning central to autism (White et al. 2010). Characterising the degree of construct overlap between autism and social anxiety can aid the development of tailored interventions to help alleviate this additional source of disability and distress.

## Social Anxiety Models

SAD is characterised by marked anxiety and fear of negative evaluation (FNE) by others in social situations and can result in social avoidance behaviour (American Psychiatric Association 2013). The cognitive model of social anxiety has been well characterised amongst neurotypical individuals (Clark an Wells 1995). Individuals with elevated social anxiety often display anticipatory worries about an upcoming social event (Clark and Wells 1995; Rapee and Heimberg 1997). They may experience an intense FNE which can lead to a negative perception of their social performance exacerbated by in-situ self-focussed attentional processes, and continued rumination over any social encounters after it has taken place (Cartwright‐Hatton et al. 2003, 2005). Overtime, continued negative social experience can lead to greater social withdrawal and avoidance, which in turn can serve to maintain symptoms of social anxiety providing temporary relief from the anxiety associated with social encounters, forming a negative cycle (Clark and Wells 1995). FNE has always been a key dimension of measurements of social anxiety (Leary 1983; Watson and Friend 1969). In an early measure of FNE developed by Watson and Friend (1969), the authors hypothesised that the degree of FNE experienced by an individual may be either related to the degree of prior social disapproval experienced, or a combination of prior social disapproval and a desire for greater social acceptance and approval.

Behavioural correlates of FNE have been studied in both autistic and neurotypical adolescents. One study found that self-reported levels of FNE predicted greater attention paid to socially threatening stimuli amongst autistic adolescents without intellectual disability, but not in neurotypical peers (White et al. 2015a, b). Another study examined eye gaze orientation patterns in neurotypical adolescents found that whereas higher level of autistic traits was related to greater latency in orienting towards the eyes, greater level of social anxiety symptoms was related to shorter latency in orienting away from the eyes (Kleberg et al. 2017). Therefore, it may be that FNE differentially affect social cognitive processing and correlate with greater social monitoring and evaluation of negative stimuli from others for autistic adolescents, and greater social avoidance and disengagement from eye contact for neurotypical adolescents.

Heightened arousal to threatening stimuli for autistic adolescents who have elevated levels of FNE may also contribute towards the development of SAD in ASD, as one mechanism proposed by Bellini (2006) suggests that autistic individuals who experience hyperarousal may show greater social withdrawal, which results in having fewer social learning opportunities over the course of development (Rubin and Burgess 2001). Autistic individuals may be more likely to be subjected to negative peer evaluation and interaction due to autism associated social impairments. Negative social experiences paired with hyperarousal and poor self-regulation might lead autistic individuals to be adversely conditioned to social exposure, and develop greater social anxiety and social withdrawal (Bellini 2006).

Others have proposed that autistic individuals who have alexithymia and poor theory of mind might in turn develop a more negative perception of their own social skills, and have greater distrust and negative perception when interacting with others (Baron-Cohen et al. 1985; Bird et al. 2010; Spain et al. 2017). In addition, other risk factors for SAD in autism include age, developmental level and cognitive abilities, and social motivation, as those who are more aware of one’s own social differences and have a desire for high quality interpersonal relationships report greater loneliness and anxiety compared to neurotypical peers (Bauminger et al. 2003; Bauminger and Kasari 2000; Kuusikko et al. 2008; Sukhodolsky et al. 2008; White et al. 2012; White and Roberson-Nay 2009). The importance of social interaction-based anxiety and social evaluative concerns as mechanisms maintaining disabling social anxiety is implicated in some, but not all of these accounts of SAD co-occurring with autism.

Nonetheless, adolescence marks a phase of social change where individuals not only begin to spend more time with peers, but peer evaluation also become more important in shaping an individual’s development of self-identity, psychological wellbeing and behaviour (Foulkes and Blakemore 2018; Gorrese and Ruggieri 2012; Lamblin et al. 2017). Social reorientation is also accompanied by neurocognitive changes during adolescence, where the time lag between the brain’s socioemotional reward system and slower maturation of the top-down cognitive-control system might make adolescents particularly sensitive to peer evaluation, and influence their decision-making in a way that is deemed to be more socially accepted and approved by their peers (Albert et al. 2013; Blakemore and Robbins 2012). The increased social exposure and heightened sensitivity to peer feedback during adolescence means that those who experience prolonged periods of social exclusion are more likely to have poorer psychological wellbeing such as increased rates of social anxiety and depression symptoms (Davis et al. 2011; Kerns and Kendall 2012; Rudolph et al. 2016).

For autistic children entering adolescence, changes in their social environment may also lead to greater awareness of their own social differences compared to neurotypical peers, especially amongst those without co-occurring intellectual disability (Deckers et al. 2017). One recent study adopted a developmental approach to investigate differences in manifestation of anxiety symptoms across younger (6–11 years) and older (12–18 years) autistic children and adolescents (Varela et al. 2019). The authors found that autistic adolescents displayed greater social evaluative concerns and social anxiety compared to their younger counterparts, suggesting that social anxiety related symptoms may become more troubling during adolescence as young people become more aware of their social differences over the course of development.

## Measurement and Construct Overlap

Although a high rate of co-occurring SAD in ASD has been documented by several studies (Spain et al. 2018; van Steensel et al. 2011; White et al. 2009), there is still considerable debate as to whether this figure is truly reflective of a condition that co-occurs alongside ASD (Wood and Gadow 2010). This issue of ‘diagnostic overshadowing’ arises when behaviours or other phenomena are attributed to the developmental diagnosis, rather than reflecting a separate and co-occurring condition (Mason and Scior 2004). Wood and Gadow (2010) described that a “true” comorbidity only occurs when the presentation of a condition (such as social anxiety) alongside autism or other developmental conditions is phenotypically identical to the same condition found in neurotypical peers.

Previous research investigating shared constructs between SAD and ASD have identified overlap in behaviours, though with important aetiological differences (Kerns and Kendall 2012; Spain et al. 2018; White et al. 2012; White, Lerner, et al. 2015a, b). There are also similarities, as both neurotypical and autistic individuals have greater negative perception and self-awareness of their social differences compared to ratings based on observations of their actual social skills (Bellini 2004; Cartwright‐Hatton et al. 2003), and experience similar changes in social anxiety across the developmental trajectory such as an increase during early childhood and adolescence (Davis et al. 2011; Kerns and Kendall 2012). In a recent study that examined common and unique autism-related anxiety experiences in autistic young people (Lau et al. 2019), social worries frequently reported included peer rejection, social differences, and lack of friendships. In addition, items from the Anxiety Scale for Children-Autism Spectrum Disorder (ASC-ASD; Rodgers et al. 2016) related to FNE and being perceived by others as different were also frequently reported. However, the original validation of the ASC-ASD scale was developed and piloted with a sample of autistic children and young people aged 8–16 years old and their parents, and therefore it remains to be explored to what extent similar constructs of social anxiety may be endorsed by older autistic adolescents above the age of 16 years using alternative social anxiety measures.

Assessing how existing questionnaires used to measure either social anxiety or autism might show construct overlap and identify behaviours unique to either SAD or ASD can achieve two goals: (1) offer some potential insight not only for clinicians seeking to use such measures; (2) further our understanding of the independence and specificity of these two conditions. In one study, White et al. (2012) used factor analysis to partition shared and unique aspects of autism and social anxiety measured by Autism Quotient (AQ; Baron-Cohen et al. 2001) and Social Phobia and Anxiety Inventory-23 (SPAI-23; Roberson-Nay et al. 2007) completed by 623 neurotypical university students (aged 18–22 years; 12 met AQ-cut off though none had clinical diagnosis of ASD). When combining items related to social difficulties from the AQ and SPAI-23, two factors relating to social anxiety and social difficulties emerged. Despite some construct overlap across social motivation, social anxiety, and social avoidance behaviours, the SPAI-23 provided a more in-depth measure of social interaction and performance-based social anxiety, while the AQ was found to capture a broader range of social difficulties and include restricted interests and preference for routine. Therefore, findings suggest that despite some common behavioural manifestations between social anxiety and autistic traits, differences in underlying mechanisms may also be evident.

However, the study had several limitations. First, although it was acknowledged that ToM and social motivation measured by the AQ could be potentially associated with social anxiety, items were not included in the factor analysis together with SPAI-23. Second, it is unclear if the findings are generalisable to individuals with an autism diagnosis. Third, given that factor analysis was only conducted between measures of autistic traits and social anxiety, it is also unclear to what extent social difficulties reported may be related to broader internalising symptoms and general wellbeing, rather than unique to social anxiety.

## Current Study

The current study aimed to replicate and extend White et al. (2012)’s findings to investigate shared latent constructs related to general social difficulty, as well as constructs uniquely related to both social anxiety, and autistic traits and symptoms. We hoped to overcome the three stated limitations of the earlier study by (i) investigating this issue in both neurotypical and autistic students; (ii) including measures of broader internalising behaviours and general wellbeing to take account of general as well as social anxiety; and (iii) including all items relating to social difficulties of the autistic trait measure in preliminary analyses to establish item redundancy. We recruited young people in both student groups as adolescence reflects a period of elevated social evaluative concerns, and thus marks a developmental phase more relevant for investigating the potential construct overlap between social anxiety and autism specific social communication differences.

## Methods

### Participants

#### Neurotypical Sample

Participants included 267 students between the ages of 17–19 years. Students were recruited from a university in the UK with a particular focus on Science, Technology, Engineering and Mathematics (STEM). Participants were recruited through campus advertisements and social media channels as part of a longitudinal study that investigated changes in students’ transition to university. Exclusion criteria include disclosure of any current diagnosis of mental, physical, medical, or other developmental conditions at the time of enrolment. Participants completed measures of social anxiety, autistic traits, and also strengths and difficulties questionnaires online via Qualtrics. Participants who completed all questionnaires were entered into a prize draw to win a £50 gift voucher or received one course credit.

#### Autism Sample

Participants included 145 students between the ages of 15–22 years. Participants were recruited through word of mouth and via social media channels and have taken part in a 3-day residential autism summer school programme held at the University of Bath, UK. All participants disclosed receiving a clinical diagnosis of autism by a clinical professional based on international criteria (American Psychiatric Association 2000, 2013) prior to attending the summer school. In cases where diagnostic information is not clear, participants were asked to show their original diagnostic letter from the clinical professional, to verify their diagnosis. Participants completed measures of social anxiety, autism symptom severity, and also a mental wellbeing measure either on paper or online via Qualtrics, prior to arriving at the summer school. Given that the summer school programme was free for all to attend, and the questionnaires were used to highlight any difficulties that students may be experiencing prior to arriving at the summer school, participants did not receive additional financial compensation for completing the questionnaires./

### Measures

#### Neurotypical Sample

##### Autism Quotient-28 (AQ-28; Hoekstra et al. 2011)

AQ-28 is an abridged version of the full 50-item AQ scale (rated on 4-point Likert scale) and has a range of items assessing social and non-social behaviours related to autistic traits. The abridged version has been validated in 3 independent samples across the Netherlands and UK. AQ-28 has good internal consistency (Cronbach’s alpha .77 to .86), and high predictive validity, where scores > 65 had a sensitivity of 0.97 and specificity of .82.

##### Social Anxiety Scale for Adolescents (SAS-A; La Greca et al 2015)

SAS-A is a 22-item (rated on 5-point Likert scale) self-report measure of social anxiety in adolescents, and forms three subscales from 18 items: (1) FNE (8 items); (2) social avoidance and distress in new situations (6 items); (3) generalised social avoidance and distress (4 items). Validation of the SAS-A in adolescents aged 15–18 years is described by La Greca et al (2015). In the current study, an independent samples t-test found no significant differences in subscale or total SAS-A scores between students > 18 years (n = 80), and students ≤ 18 years (n = 187). Given all students experienced similar social pressures during the first two weeks of transition to university, we did not expect significant age-related differences in students’ ability to adapt to the social environment.

##### Strengths and Difficulties Questionnaire 18 + (SDQ; Goodman et al. 1998)

SDQ 18 + version is a 25-item (rated on 3-point Likert scale) self-report measure of both internalising and externalising behaviours experienced by individuals aged 18 and above, including emotional symptoms, conduct problems, hyperactivity and/or inattention, peer relationship problems, and prosocial behaviour. The self-report version of the SDQ has good internal reliability, with Cronbach’s alpha being 0.61 to 0.82 for the different subscales and total scores.

### Autism Sample

#### Social Responsiveness Scale—Short (SRS-S; Kanne et al. 2009)

The SRS-S is an 11-item (rated on 4-point Likert scale) self-report measure of autism symptom severity developed from the full SRS-S (Constantino and Gruber 2005, 2012). The SRS-S has been used in research with both adults (Wakeford et al. 2015) and adolescents (Brosnan et al. 2014). The short version has been validated against the full SRS-S (Kanne et al. 2009). The SRS-S was used to measure self-perceived level of autism symptom severity in the current study, and not as a verification of autism diagnosis.

#### Social Anxiety Scale for Adolescents (SAS-A; La Greca et al 2015)

See neurotypical sample above for measure description. In the current study, an independent samples t-test found no significant differences in subscale or total SAS-A scores between students > 18 years (n = 21), and students ≤ 18 years (n = 124), indicating similar levels of social anxiety reported whether students were above or below 18 years old.

#### Warwick Edinburgh Mental Wellbeing Scale (WEMWBS; Tennant et al. 2007)

WEMWBS is a 14-item (rated on 5-point Likert scale) self-report measure of both mental health and positive affect, as well as interpersonal relationships and positive functioning. WEMWBS has good internal consistency (Cronbach’s alpha > .70) and showed good criterion validity with strong correlations with other measures of positive affect/wellbeing, and some measures of physical and mental health outcomes.

### Data Analyses

All statistical analyses were completed using SPSS v25 (IBM SPSS Statistics 2016). We first examined the psychometric properties (data distribution and internal consistency) of all questionnaire measures. We computed an exploratory factor analysis of both AQ-28 and SRS-S, to examine which items in each scale more specifically related to social difficulties. Next, in neurotypical sample, we conducted an exploratory factor analysis between items related to social difficulties from AQ-28, SAS-A, and the internalising subscale of the SDQ. In autism sample, we conducted an exploratory factor analysis between items related to social difficulties from the SRS-S, SAS-A, and items related to social wellbeing and relationship from WEMWBS identified from an exploratory factor analysis. All factor analyses used oblique (direct oblimin) rotation due to potential overlap and correlation across different factors. Scree plot and an eigenvalue of 1.0 or higher were used to select factors retained in the final model. Each factor was examined to see whether the items loaded onto that factor were related conceptually and theoretically to each other. Each item was examined to explore whether it loaded saliently (loading ≥ .40 for any factor) and uniquely (loading of ≥ .40 for one factor only) onto each factor. Next, we conducted bivariate correlations to assess whether the relationship between autistic traits/autism symptom severity and social anxiety might be stronger than that predicted by shared method variance (i.e., using self-reports). Finally, we compared differences in autistic traits/autism symptom severity between students who had high levels of social anxiety (i.e., above recommended clinical cut-off score on SAS-A), amongst neurotypical and autistic students.

## Results

### Neurotypical Sample

Participant demographic information and measurement scores are shown in Table 1. 61 (22.8%) participants had an AQ-28 score of ≥ 70, indicating elevated level of autistic traits (Hoekstra et al. 2011). Of the 61 students, 60 also had a SAS-A total score above the cut-off threshold (i.e., > 50), showing a very high level of co-occurrence between elevated autistic traits and social anxiety symptoms. Overall, a total of 174 students (65% of the sample) scored above the cut-off score at 50 for SAS-A and showed elevated levels of social anxiety symptoms.

**Table 1:** Neurotypical sample (n = 267)—demographic information

	M (SD)	Range	Skewness	Kurtosis	Cronbach’s α
Age	18.28 (.50)	17–19	.43	− .57	–
A-Level average	5.08 (.58)	3–6	–	–	–
Gender	(n)	(%)		–	–
Male	54	20.20	–	–	–
Female	213	79.80	–	–	–
Unknown	0	0	–	–	–
Ethnicity					
White	211	79.00	–	–	–
Black	3	1.12	–	–	–
Asian	37	13.86	–	–	–
Mixed/other	16	5.99	–	–	–
Faculty			–	–	
Science	33	16.50	–	–	–
Engineering	13	4.90	–	–	–
Technology	11	4.10	–	–	–
Mathematics	6	2.20	–	–	–
Humanities and arts	9	3.40	–	–	–
Social sciences	184	68.90	–	–	–
AQ-28					
Social behaviour	54.63 (8.88)	32–83	.30	.21	.79
Number/pattern	11.21 (3.21)	5–20	.29	− .19	.73
Total	62.72 (9.90)	36–92	.30	.22	.83
SAS-A					
Fear of negative evaluation	25.07 (6.18)	9–40	.13	− .05	.90
New situations	20.01 (4.49)	8–30	.09	− .32	.86
General	10.93 (3.21)	4–20	.28	− .01	.77
Total	56.02 (11.94)	23—87	.19	− .05	.92
SDQ					
Internalising	7.11 (3.16)	0–18	.28	− .08	.67; .69*
Externalising	5.06 (2.59)	0–13	.45	.19	.57
Total	12.17 (4.54)	0–28	.41	.43	.60

*AQ-28* autism quotient 28, *SAS-A* social anxiety scale for adolescents, *SDQ* strengths and difficulties questionnaire

*Internal consistency after removing item 14 (other people generally like me) and item 23 (I get along better with older people than with people of my own age) from scale. Both items showed a corrected item-total correlation of < .30 (.16 and .15 respectively)

We conducted an exploratory factor analysis using oblique rotation (direct oblimin) on the 28 items of the AQ-28 (see Appendix 1). Seven factors were identified which explained 56.31% of the variance and related to: (1) social difficulties; (2) imagination; (3) fascination with numbers/patterns; (4) task switching and attention; (5) preference for routine; (6) theory of mind; (7) social motivation. This model is similar to that reported by Hoekstra et al. (2011), the only difference being items from the AQ-28 in relation to Theory of Mind loaded onto a distinct factor which described social cognition differences when understanding mental states of others and was independent of items that related to broader social communication difficulties.

We conducted an exploratory factor analysis between items identified from the social difficulties (factor 1; 5 items), imagination (factor 2; 3 items), theory of mind (factor 6; 3 items), and social motivation (factor 7; 3 items) from the AQ-28, as well as the SAS-A, and also the internalising subscales of the SDQ (with items 14 and 23 removed, see Table 1), which contained items that assessed students’ emotional difficulties and peer relationships. Using oblique rotation (direct oblimin), the Kaiser–Meyer–Olkin verified the sampling adequacy for the analysis, KMO = 0.92, which is above the acceptable limit of 0.5 (Kaiser and Rice 1974). An initial analysis was run to obtain eigenvalues for each factor in the data. Seven factors emerged with eigenvalues greater than one and together accounted for 61.99% of the variance in the data. Table 2 shows both the structure and pattern matrices revealing cross-loadings as well as unique relationship between each item and the factors identified. Across both matrices, items in relation to social distress in new situations (Factor One) showed construct overlap across all three measures (AQ-28, SAS-A, and SDQ). In contrast, items from the AQ-28 and SAS-A also reflected unique dimensions of broader social communication skills, and items from the SDQ reflected mental and physical wellbeing beyond that of social anxiety. In terms of variance, the pattern matrix indicated that the first factor (social distress in new situations; items from AQ-28, SDQ, SAS-A) accounted for 31.59%; factor two (FNE; items from SAS-A) accounted for 9.89%; factor three (Theory of Mind difficulties; items from AQ-28) accounted for 5.14%; factor four (Social motivation; items from AQ-28 and SDQ) accounted for 4.78%; factor five (mental and physical wellbeing; items from SDQ and AQ-28) accounted for 4.36%; factor six (relationship worries; items from SDQ and SAS-A) accounted for 3.39%; and factor seven (bullying; item from SDQ) accounted for 2.84%.

**Table 2:** Neurotypical sample (n = 267)—Factor loadings of exploratory factor analysis (social difficulties, social anxiety, and internalising symptoms)

Item	Measure	F1	F2	F3	F4	F5	F6	F7
(a) Structure matrix								
I find social situations easy	AQ-28	**.813**	− .302	.288	**.412**	.152	− .246	− .101
I feel shy around people I don’t know	SAS-A	**.786**	− .344	.294	.294	.284	− .155	.072
I get nervous when I talk to peers I don’t know very well	SAS-A	**.777**	− **.490**	.269	.224	.360	− .236	.079
I’m quiet when I’m with a group of people	SAS-A	**.771**	− .265	.152	.351	.124	− .322	.058
I find it hard to make new friends	AQ-28	**.747**	− .317	.339	.341	.206	− .267	− .139
I get nervous when I meet new people	SAS-A	**.745**	− **.497**	.303	.194	**.440**	− .228	.000
I am nervous in new situations. I easily lose confidence	SDQ	**.719**	− .394	.186	.175	**.431**	− .140	.243
I only talk to people I know really well	SAS-A	**.715**	− .193	.240	.261	.049	− .248	− .102
I enjoy meeting new people	AQ-28	**.642**	− .199	.108	**.510**	.071	− .171	− .129
I feel nervous when I’m around certain people	SAS-A	**.601**	− **.532**	.047	.171	.328	− **.502**	.001
I worry about what others say about me	SAS-A	.278	− **.854**	.130	.024	.330	− .221	.008
I worry that others don’t like me	SAS-A	.396	− **.828**	.048	.122	.334	− .241	.011
I worry about what others think of me	SAS-A	.328	− **.818**	.047	.071	.360	− .086	.216
I’m afraid that others will not like me	SAS-A	.388	− **.813**	.082	.037	.303	− .169	.130
I feel that others make fun of me	SAS-A	.201	− **.719**	.197	.158	.253	− .297	− .378
I worry about being teased	SAS-A	.374	− **.708**	.125	.088	.261	− .090	− .184
If I get into an argument, I worry that the other person will not like me	SAS-A	.195	− **.663**	.048	− .032	.161	− .331	− .001
I feel that peers talk about me behind my back	SAS-A	.303	− **.652**	.169	.158	.178	− .269	− .347
I worry about doing something new in front of others	SAS-A	.501	− **.543**	.241	.177	.395	− .059	.194
I find it difficult to work out people’s intentions	AQ-28	.282	− .224	**.822**	.004	.118	− .272	− .134
When I’m reading a story, I find it difficult to work out the character’s intentions	AQ-28	.123	− .008	**.776**	.051	.059	− .113	− .023
I find it easy to work out what someone is thinking or feeling just by looking at their face	AQ-28	.267	− .087	**.713**	.219	− .092	− .076	.044
I prefer to do things with others rather than on my own	AQ-28	.218	− .049	− .022	**.782**	.015	− .218	− .038
I would rather be alone than with other people	SDQ	.364	− .105	.050	**.740**	.251	− .264	− .108
I find myself drawn more strongly to other people than do things	AQ-28	.292	− .088	.300	**.701**	− .065	− .010	− .044
I enjoy social occasions	AQ-28	.**610**	− .113	.223	**.660**	.071	− .304	− .159
I worry a lot	SDQ	.130	− **.458**	− .016	.059	**.737**	− .114	.223
I am often unhappy, depressed, or tearful	SDQ	.244	− .311	.073	.195	**.675**	− .329	− .295
New situations make me anxious	AQ-28	**.600**	− .357	.121	.100	**.637**	− .131	.104
I get a lot of headaches, stomach aches or sickness	SDQ	.285	− .131	.053	.000	**.625**	− .072	− .288
I have many fears, I am easily scared	SDQ	.190	− **.481**	.125	.062	**.582**	− .181	− .118
I feel shy even with peers I know well	SAS-A	**.430**	− .344	.277	.269	.216	− **.680**	− .070
It’s hard for me to ask others to do things for me	SAS-A	**.434**	− **.415**	.072	.265	.034	− **.675**	.195
I’m afraid to invite others to do things with me because they might say no	SAS-A	**.502**	− **.501**	.167	.070	.137	− **.664**	− .109
I have at least one good friend	SDQ	.051	.021	.260	.205	.153	− **.581**	− .154
Other people pick on me or bully me	SDQ	.110	− .240	.039	.166	.298	− .095	− **.688**
(b) Pattern matrix								
I find social situations easy	AQ-28	**.738**	− .029	.088	.154	− .041	− .024	− .086
I’m quiet when I’m with a group of people	SAS-A	**.726**	.013	− .042	.106	− .050	− .156	.073
I only talk to people I know really well	SAS-A	**.724**	.053	.064	.006	− .116	− .084	− .101
I feel shy around people I don’t know	SAS-A	**.703**	− .058	.125	.064	.110	.054	.083
I find it hard to make new friends	AQ-28	**.649**	− .046	.157	.092	.021	− .053	− .117
I get nervous when I talk to peers I don’t know very well	SAS-A	**.647**	− .201	.095	− .007	.151	− .021	.094
I am nervous in new situations. I easily lose confidence	SDQ	**.625**	− .093	.042	− .016	.279	.034	.256
I get nervous when I meet new people	SAS-A	**.599**	− .197	.137	− .033	.238	− .007	.021
I enjoy meeting new people	AQ-28	**.571**	− .009	-.068	.331	− .073	.017	− .115
I feel nervous when I’m around certain people	SAS-A	**.435**	− .279	− .130	− .032	.113	− .352	.033
I worry about what others say about me	SAS-A	− .045	− **.836**	.051	− .027	.074	− .034	.026
I worry about what others think of me	SAS-A	.032	− **.791**	− .026	.047	.129	.097	.225
I worry that others don’t like me	SAS-A	.102	− **.765**	− .068	.043	.070	− .040	.026
I’m afraid that others will not like me	SAS-A	.126	− **.762**	− .013	− .036	.048	.017	.135
I feel that others make fun of me	SAS-A	− .130	− **.717**	.104	.095	.017	− .097	− .344
I worry about being teased	SAS-A	.159	− **.670**	.023	− .002	.016	.123	− .188
If I get into an argument, I worry that the other person will not like me	SAS-A	− .039	− **.653**	− .025	− .093	− .054	− .220	.016
I feel that peers talk about me behind my back	SAS-A	.051	− **.625**	.057	.057	− .063	− .070	− .329
I worry about doing something new in front of others	SAS-A	.285	− .383	.147	.078	.228	.132	.213
When I’m reading a story, I find it difficult to work out the character’s intentions	AQ-28	− .042	.082	**.794**	− .034	.055	− .036	.034
I find it difficult to work out people’s intentions	AQ-28	.083	− .083	**.788**	− .158	.020	− .144	− .079
I find it easy to work out what someone is thinking or feeling just by looking at their face	AQ-28	.103	− .031	**.690**	.119	− .151	.046	.086
I prefer to do things with others rather than on my own	AQ-28	− .028	− .002	− .126	**.791**	− .008	− .105	.032
I would rather be alone than with other people	SDQ	.111	.061	− .083	**.689**	.212	− .15	− .031
I find myself drawn more strongly to other people than do things	AQ-28	.061	− .075	.222	**.682**	− .118	.159	.006
I enjoy social occasions	AQ-28	**.463**	.102	.044	**.485**	− .040	− .120	− .109
I worry a lot	SDQ	− .157	− .290	− .040	.093	**.694**	− .026	.278
I am often unhappy, depressed, or tearful	SDQ	.005	− .062	− .022	.116	**.615**	.206	− .227
I get a lot of headaches, stomach aches or sickness	SDQ	.241	.132	− .019	− .103	**.602**	.026	− .266
New situations make me anxious	AQ-28	**.497**	− .026	− .003	− .062	**.529**	.024	.127
I have many fears, I am easily scared	SDQ	− .071	− .335	.070	.025	**.479**	− .048	− .072
It’s hard for me to ask others to do things for me	SAS-A	.219	− .251	− .077	.109	− .144	− **.600**	.253
I have at least one good friend	SDQ	− .143	.183	.205	.123	.161	− **.582**	− .061
I feel shy even with peers I know well	SAS-A	.186	− .108	.131	.084	.069	− **.573**	.009
I’m afraid to invite others to do things with me because they might say no	SAS-A	.342	− .293	.001	− .158	− .092	− **.549**	− .071
Other people pick on me or bully me	SDQ	.009	− .176	− .037	.050	.203	.042	− **.676**

*AQ-28* autism quotient-28, *SAS-A* social anxiety scale for adolescents, *SDQ* strengths and difficulties questionnaire, *F1* Social distress in new situations, *F2* Fear of negative evaluation, *F3* Theory of mind difficulties, *F4* Social motivation, *F5* Mental and physical wellbeing, *F6* Relationship worries, *F7* Bullying

Bold values are statistically significant

Using bivariate correlations, we found that AQ-28 and SAS-A total scores significantly correlated with each other (*r* = .59, *p* < .001). Both AQ-28 and SAS-A also significantly correlated with the SDQ (*r* = .47, *r* = .54, respectively, *p* < .001 for both). Using Fisher’s *R* to *Z* transformations, the correlation between AQ-28 and SAS-A (*z′* = .68) was significantly stronger than with SDQ (*z′* = .51, .56 respectively). Therefore, the shared variance between AQ-28 and SAS-A withstands beyond that of shared method variance by using self-reports across all three measures. See Appendix 2 for further comparison of autistic symptom severity between neurotypical students who reported low versus high levels of social anxiety.

### Autism Sample

Participants’ demographic and measurement information are shown in Table 3. A total of 109 students (75% of the sample) scored above the cut-off score at 50 for SAS-A and showed elevated levels of social anxiety symptoms.

**Table 3:** Autism sample (n = 145)—demographic information

	M (SD)	Range	Skewness	Kurtosis	Cronbach’s α
Age	17.59 (1.1)	15–22	1.60	4.49	–
Gender	(n)	(%)			
Male	99	68.3	–	–	–
Female	43	29.7	–	–	–
Unknown	3	2.1	–	–	–
Ethnicity					
White	132	91.03	–	–	–
Asian	4	2.76	–	–	–
Black	1	069	–	–	–
Mixed/other	7	4.83	–	–	–
SAS-A total	59.99 (13.97)	22–89	− .13	− .35	.93
Fear of negative evaluation	25.52 (7.86)	8–40	− .14	− .51	.92
Social avoidance/distress (New)	21.89 (4.93)	7–30	− .47	− .35	.86
Social avoidance/distress (Gen)	12.57 (3.52)	4–20	− .02	− .49	.74
SRS total	18.20 (5.95)	0–32	− .25	.08	.80
WEMWBS total	44.52 (8.08)	19–70	− .05	.63	.86

*SAS-A* social anxiety scale for adolescents, *Gen* general, *SRS* social responsiveness scale, *WEMWBS* Warwick Edinburgh Mental Wellbeing Scale

We conducted two exploratory factor analyses using oblique rotation (direct oblimin) separately on the 11 items of the SRS-S (see Appendix 1), and on the 14 items of the WEMWBS (see Appendix 3). For the SRS-S, three factors were identified which explained 53.63% of the variance and related to: (1) social difficulties; (2) social motivation; (3) sensory and other difficulties. For WEMWBS, four factors were identified which explained 61.58% of the variance and related to: (1) social wellbeing and relationships; (2) decision making/problem solving; (3) mental wellbeing; (4) self-esteem and future.

Next, we conducted an exploratory factor analysis between items from the social difficulties (Factor 1; 5 items) and social motivation (Factor 2; 1 item) of the SRS-S, as well as the SAS-A (18 items), and also the social wellbeing and relationships (Factor 1; 5 items) of the WEMWBS. Using oblique rotation (direct oblimin), the Kaiser–Meyer–Olkin verified the sampling adequacy for the analysis, KMO = .87, which is above the acceptable limit of 0.5 (Kaiser and Rice 1974). An initial analysis was run to obtain eigenvalues for each factor in the data. Six factors emerged with eigenvalues greater than one and together accounted for 64.46% of the variance in the data. Given the significant overlap in factor loadings across the factors, we have chosen to report both the structure and pattern matrices in Table 4 to show both cross-loadings as well as unique relationships between each item and the factors identified. In terms of variance, the pattern matrix indicated that the first factor (FNE; items from SAS-A) accounted for 31.93%; factor two (social wellbeing and relationships; items from WEMWBS) accounted for 11.21%; factor three (social distress in new situations; items from SAS-A) accounted for 7.11%; factor four (autism specific social difficulties; items from SRS-S) accounted for 6%; factor five (social motivation; item from SRS-S) accounted for 4.19%; and factor six (peer relationships; items from SAS-A and SRS-S) accounted for 4.02%.

**Table 4:** Autism sample (n = 145)—factor loadings of exploratory factor analysis (social difficulties, social anxiety, and internalising symptoms)

Item	Measure	F1	F2	F3	F4	F5	F6
(a) Structure matrix							
I worry about what others say about me	SAS-A	**.840**	− .117	.351	.167	− .317	− .380
I worry that others don’t like me	SAS-A	**.831**	− .168	**.464**	.200	− .293	− **.494**
I feel that others make fun of me	SAS-A	**.813**	− .072	.308	.327	.173	− .211
I feel that peers talk about me behind my back	SAS-A	**.800**	− .142	.131	.127	.205	− .227
I worry about what others think of me	SAS-A	**.791**	− .195	.316	.068	− .369	− **.420**
I’m afraid that others will not like me	SAS-A	**.777**	− .272	.363	.183	− .308	− **.442**
I worry about being teased	SAS-A	**.759**	− .063	**.445**	.167	− .033	− .246
I feel nervous when I’m around certain people	SAS-A	**.652**	− .080	**.493**	.271	.049	− **.487**
If I get into an argument, I worry that the other person will not like me	SAS-A	**.563**	− .081	.296	.148	− .249	− **.545**
I worry about doing something new in front of others	SAS-A	**.519**	.035	**.465**	.268	− .153	− **.434**
I’ve been feeling good about myself	WEMWBS	− .166	**.830**	− .216	− .035	− .057	.253
I’ve been feeling cheerful	WEMWBS	− .131	**.771**	− .123	− .006	− .135	.163
I’ve been feeling useful	WEMWBS	− .098	**.755**	− .242	− .328	− .110	.114
I’ve been feeling close to other people	WEMWBS	− .049	**.727**	− .245	− .294	− .261	.058
I feel shy around people I don’t know	SAS-A	.334	− .232	**.869**	.250	− .026	− .294
I get nervous when I talk to peers I don’t know very well	SAS-A	.381	− .223	**.862**	.225	− .057	− **.434**
I get nervous when I meet new people	SAS-A	.370	− .208	**.838**	.249	.019	− .328
I only talk to people I know really well	SAS-A	.145	− .183	**.768**	.206	.082	− .334
I’m quiet when I’m with a group of people	SAS-A	.190	− .198	**.670**	.215	.172	− **.460**
I have difficulty relating to peers	SRS	.206	− .231	.114	**.733**	.203	− .394
Compared to others, I have a restricted or unusually narrow range of interests	SRS	.027	− .122	.246	**.692**	.004	− .163
I am sometimes regarded by other people as odd or weird	SRS	.338	− .011	.143	**.686**	.339	.088
I have trouble keeping up with the flow of a normal conversation	SRS	.242	− .205	.389	**.636**	− .064	− .279
I would rather be alone than with others	SRS	.099	− .253	.210	.165	**.773**	− .245
I’ve been feeling interested in other people	WEMWBS	.163	**.490**	.090	− .129	− **.652**	− .079
It’s hard for me to ask others to do things with me	SAS-A	.226	− .237	**.427**	.231	.216	− **.755**
I have difficulty making friends, even when trying my best	SRS	.387	− .141	.399	**.424**	.158	− **.709**
I’m afraid to invite others to do things with me because they might say no	SAS-A	.352	− .195	.**413**	.195	− .052	− **.707**
I feel shy even with peers I know well	SAS-A	.351	− .119	**.524**	.225	.029	− **.596**
(b) Pattern matrix							
I feel that peers talk about me behind my back	SAS-A	**.875**	− .063	− .153	− .052	.280	− .007
I feel that others make fun of me	SAS-A	**.821**	.047	.052	.146	.226	.091
I worry about what others say about me	SAS-A	**.761**	− .058	.059	.018	− .262	− .089
I worry about being teased	SAS-A	**.712**	.039	.264	− .023	.032	.081
I worry about what others think of me	SAS-A	**.707**	− .158	.017	− .079	− .316	− .162
I worry that others don’t like me	SAS-A	**.691**	− .077	.151	.018	− .246	− .186
I’m afraid that others will not like me	SAS-A	**.664**	− .215	.040	.025	− .283	− .161
I feel nervous when I’m around certain people	SAS-A	**.503**	.075	.237	.068	.096	− .231
If I get into an argument, I worry that the other person will not like me	SAS-A	**.410**	− .001	.000	.025	− .209	− **.402**
I worry about doing something new in front of others	SAS-A	.342	.157	.268	.131	− .118	− .216
I’ve been feeling good about myself	WEMWBS	− .076	**.824**	− .015	.109	.037	.097
I’ve been feeling cheerful	WEMWBS	− .094	**.775**	.048	.120	− .049	.038
I’ve been feeling useful	WEMWBS	.009	**.728**	− .072	− .231	.031	− .083
I’ve been feeling close to other people	WEMWBS	.030	**.685**	− .123	− .180	− .130	− .132
I feel shy around people I don’t know	SAS-A	.074	− .072	**.862**	.026	− .044	.095
I get nervous when I meet new people	SAS-A	.122	− .039	**.804**	.019	.012	.045
I get nervous when I talk to peers I don’t know very well	SAS-A	.095	− .052	**.794**	− .011	− .061	− .076
I only talk to people I know really well	SAS-A	− .120	− .017	**.777**	.007	.062	− .058
I’m quiet when I’m with a group of people	SAS-A	− .066	− .019	**.586**	.006	.162	− .245
Compared to others I have a restricted or unusually narrow range of interests	SRS	− .165	− .030	.095	**.704**	− .114	− .043
I have difficulty relating to peers	SRS	.036	− .106	− .234	**.697**	.102	− .333
I am sometimes regarded by other people as odd or weird	SRS	.341	.096	.002	**.646**	.285	.295
I have trouble keeping up with the flow of a normal conversation	SRS	.027	− .096	.185	**.579**	− .157	− .070
I would rather be alone than with others	SRS	.071	− .088	.093	− .028	**.773**	− .190
I’ve been feeling interested in other people	WEMWBS	.182	**.404**	− .034	− .007	− **.578**	.064
It’s hard for me to ask others to do things with me	SAS-A	− .040	− .054	.135	.039	.213	− **.702**
I’m afraid to invite others to do things with me because they might say no	SAS-A	.101	− .057	.116	.034	− .044	− **.610**
I have difficulty making friends, even when trying my best	SRS	.140	.046	.051	.259	.152	− **.607**
I feel shy even with peers I know well	SAS-A	.108	.042	.311	.044	.044	− **.436**

*SAS-A* social anxiety scale for adolescents, *SRS* social responsiveness scale, *WEMWBS* Warwick Edinburgh Mental Wellbeing Scale, *F1* Fear of negative evaluation, *F2* Social wellbeing and relationships, *F3* Social distress in new situations, *F4* Autism specific social difficulties,  *F5* Social motivation, *F6* Peer relationships

Bold values are statistically significant

Using bivariate correlations, we found that SRS-S and SAS-A total scores significantly correlated with each other (*r* = .54, *p* < .001). Both SRS-S and SAS-A also significantly correlated with the WEMWBS (*r* =  − .42, *r* =  − .40, respectively, *p* < .001 for both). Using Fisher’s *R* to *Z* transformations, the correlation between SRS-S and SAS-A was significantly stronger than with WEMWBS. Therefore, the shared variance between SRS-S and SAS-A withstands beyond that of shared method variance by using self-reports across all three measures. See Appendix 2 for further comparison of autism symptom severity between autistic students who reported low versus high levels of social anxiety. A comparison of the FNE factor from neurotypical and autism samples and that of the original subscale in SAS-A is shown in Table 5.

**Table 5:** Comparison of fear of negative evaluation subscale items from social anxiety scale for Adolescents and those derived from factor analysis in neurotypical and autistic student samples

Item (number)	La Greca and Lopez (1998)	Neurotypical sample	Autism sample
I worry about what others say about me (12)	Y	Y	Y
I worry that others don’t like me (14)	Y	Y	Y
I’m afraid that others will not like me (9)	Y	Y	Y
I worry about what others think of me (8)	Y	Y	Y
I feel that others make fun of me (17)	Y	Y	Y
I worry about being teased (3)	Y	Y	Y
I feel that peers talk about me behind my back (6)	Y	Y	Y
If I get into an argument, I worry that the other person will not like me (8)	Y	Y	Y
I worry about doing something new in front of others (1)	N (Social avoidance and distress – New situations)	Y*	Y*
I feel nervous when I’m around certain people (20)	N (Social avoidance and distress – New situations)	Y*	Y

*Structure matrix only

## Discussion

The current study aimed to replicate and extend White et al. (2012)’s findings to identify shared and unique aspects of autism and social anxiety measured by validated standardised self-report measures in both neurotypical and autistic students. Next, we discuss our findings on construct overlap and how they inform both our conceptual understanding of social anxiety in the context of autism and also implications for clinical practice, before highlighting study limitations and future directions.

### Examining Construct Overlap

#### Social Anxiety Symptoms

We extended White et al.’s (2012) investigation by including autistic as well as non-autistic (neurotypical) participants and found that FNE was a distinct factor related to social anxiety across both samples. Closer inspection of the items which loaded on to the FNE factor from both samples matched closely to the FNE subscale from the social anxiety measure (SAS-A) for both neurotypical and autistic students. Our findings suggest that FNE as measured by SAS-A is a relatively stable construct underlying social anxiety in both neurotypical and autistic students and is qualitatively distinct from other social communication difficulties related to autism. Our study design also allowed us to address a second limitation from the White et al.’s (2012) study by including items relating to general emotional wellbeing and other internalising symptoms from standardised measures (WEMWBS and SDQ), with the purpose of exploring to what extent social difficulties reported by both groups may be related to broader internalising symptoms and general wellbeing rather than unique to social anxiety. Items from both the WEMWBS and SDQ were also distinct to those items reporting on FNE, thus further highlighting how FNE might be a key underlying or maintaining factor that can increase an individual’s vulnerability specifically to social anxiety.

Evidence supporting construct invariance of FNE as measured by SAS-A is also concordant with one recent study that examined psychometric properties of the SAS-A when completed by autistic youths and their caregivers (Schiltz et al. 2019). Compared to items that loaded onto the SAD factor which described both generalised social anxiety and anxiety in new social situations, the magnitude of the FNE subscale’s factor loadings showed greater consistency between autistic young people and their caregivers. Therefore, results support FNE measured by SAS-A to reflect a more stable psychological construct that has greater measurement invariance across different informants when used in an autism sample. This is especially encouraging given that the primary purpose of the current study was to explore the unique and shared constructs underlying social difficulties captured by both measures of autistic traits/symptoms, and social anxiety, in a way that is comparable across both autistic and TD individuals. Therefore, our findings are in line with that of Schiltz et al. (2019)’s analysis of the psychometric properties of SAS-A, to highlight that it consistently captures FNE across both autistic and neurotypical young people.

However, it should be noted that beyond FNE, the manifestation of social anxiety in autism may present unique symptoms that are qualitatively distinct from neurotypical peers. Development and use of autism specific measures of social anxiety may be clinically useful for its assessment and diagnosis in clinical settings (Kreiser and White 2014). One example is the Social Anxiety Scale for People with ASD (SASPA; Kreiser and White 2011) which was developed by integrating expert opinions from both autism and anxiety disorders specialists, and behaviours exhibited by autistic individuals who experience co-occurring symptoms of social anxiety. Although further psychometric analysis of both validity and reliability of the SASPA measure is needed (Kreiser and White 2014), it does offer an important first step towards highlighting unique symptoms of social anxiety when co-occurring alongside autism that can supplement more traditional measures when used in a clinical setting, to overcome potential challenges of diagnostic overshadowing when working with autistic individuals.

#### Autism Symptoms

We also addressed a third limitation from White et al. (2012)’s study by including items from the AQ-28 related to theory of mind and social motivation in our factor analysis for neurotypical students, as both factors may be potentially associated with social anxiety. We found that only items relating to theory of mind difficulties as measured by AQ-28 were distinctly related to autistic traits and did not cross-load onto other factors relating social relationships and social distress. Items from the AQ-28 measuring social communication difficulties related to autistic traits in neurotypical students showed construct overlap with measures of social anxiety and broader internalising symptoms. In contrast, social communication difficulties measured by SRS-S amongst autistic students loaded more distinctly onto a latent factor specifically highlighting social challenges related to autism, rather than social anxiety and general wellbeing. The correlation between social anxiety and autistic traits/symptom severity was stronger in both neurotypical and autistic students compared to that with general wellbeing and broader internalising symptoms. Furthermore, students who were more socially anxious also exhibited higher levels of non-socially related autistic traits. Therefore, the shared variance between measures of social anxiety and autistic traits are neither simply a result of shared methodology variance (i.e., by using self-report measures), nor is it simply a consequence of shared social communication difficulties. Our findings thus replicate and support White et al. (2012)’s notion that social anxiety and autism do not merely reflect measurement error relating to diagnostic overlap. Instead, the two conditions can be conceptualised as ‘true comorbidity’ as defined by Caron and Rutter (1991), as they may share certain risk factors (whether biological or environmental) and that there might be some degree of reciprocal relationship such that the presence of one condition might exacerbate the development or manifestation of the other.

It should be noted that we observed elevated level of autistic traits in our neurotypical sample, which has a female majority (80%). One study by Abu-Akel et al. (2019) highlighted that the cut-off score of the AQ may vary across clinical and non-clinical subpopulations, with one example being that the mean AQ score is lower for autistic and non-autistic females compared to their male counterparts, yet the cut-off score is higher for females compared to males. This finding is in line with the female protective effect which suggests that in order for females to exhibit similar levels of autistic traits as males, they must carry relatively greater genetic load and neurobiological differences (Lei et al. 2019; Robinson et al. 2013). It may be that the higher level of autistic traits observed in the current sample of neurotypical students reflect the nature of the sample being selected from a STEM-based university (27.7% studied STEM; 68.9% studied social sciences), where elevated rates of autistic traits have been observed in the neurotypical population (Ruzich et al. 2015). Although a majority of our neurotypical sample have a social sciences background and were female, given that they are studying at an academically competitive STEM university in the UK, the standard entry criteria would include high academic performance in basic sciences and mathematics compared to social sciences students enrolled in a non-STEM specific university. Future studies may thus investigate to what extent sex differences in level of autistic traits identified from Abu-Akel et al. (2019)’s study may be generalisable when applied to those working or studying in STEM disciplines compared to humanities subjects, and whether higher cut-off scores may be warranted for such sub-populations in relation to autism diagnosis.

Another potential reason accounting for elevated autistic traits on the AQ may be attributed to the construct overlap between autistic traits and social anxiety in the neurotypical sample, given that 60 out of the 61 students who scored above the AQ cut-off also scored above the clinical cut-off on the SAS-A. Tonge et al. (2016) found that compared to a sample of neurotypical adults, non-autistic adults who had a clinical diagnosis of generalised social anxiety disorder (GSAD) scored higher on the AQ-Short, which was largely accounted by elevated scores on the social skills subscale. The authors argued that items such as *I find it hard to make friends* and *I find social situations easy* from the social skills subscale characterised complaints social interaction difficulties, rather than genuine social skills deficits. The phrasing of items using relative comparison words such as “hard” or “easy” may be more likely to elicit negative self-bias during social comparisons for those who experienced heightened social anxiety compared to their non anxious peers.

In the current study, such items form the AQ also showed greater construct overlap with those from the social anxiety scale and loaded onto a factor that reflected general distress in new situations for neurotypical students. Therefore, for some neurotypical students who experienced greater social anxiety, it may be that shared complaints of social interaction difficulties rather than social skills deficits led to an increase in social aspects of autistic traits captured by the AQ. However, there may be qualitative differences in the nature of construct overlap between autistic traits and social anxiety between clinical and non-clinical populations, especially when disentangling the role of social and non-social aspects of autistic traits. It may be helpful for future studies to also include a sample with a clinical diagnosis of SAD for comparison in addition to an autism group and neurotypical sample and assess generalisability of current findings.

### Conceptual Understanding

FNE identified as a relatively stable construct in relation to social anxiety across both samples and might serve as an important mechanism in the development and maintenance of SAD regardless of autism diagnosis. In our study, elevated levels of FNE for both student groups reflected a shared common mechanism underlying social anxiety. Regardless of autism diagnosis, adolescents facing an acute stressor that result in changes in their immediate social environment want to be accepted by their new peers, and do not want to be rated as socially awkward or be otherwise disapproved of. Such construct invariance provides some face validity and generalisability for using the SAD model developed in neurotypical population as proposed by Clark and Wells (1995) to better understand social anxiety in the context of autism. When interpreted alongside autism specific social anxiety models, hyperarousal and increased sensitivity to negative evaluation by others may contribute towards autistic adolescents to perceive aversive social interactions as more pervasive and prevalent over time, and further increase social anxiety and fears of social disapproval (Bauminger et al. 2003; Bellini 2006; White et al. 2012; White and Roberson-Nay 2009). Such a negative cycle may be especially detrimental for autistic students with greater levels of sociability and social motivation (Cheek and Buss 1981; Chevallier et al. 2012), who may be even more likely to encounter and be sensitive to negative social feedback during the process of actively seeking and developing one’s peer network.

### Clinical Implications

Our findings highlight that FNE is a key component of social anxiety co-occurring with autism. How best to adapt the evidence-based CBT treatment protocols for social anxiety so that the cognitive techniques with proven efficacy are accessible to autistic individuals is a critical question for clinical research. Well documented difficulties in noticing and reporting subtle shifts in emotional and bodily states (Hill et al. 2004; Rieffe et al. 2011; Roberts-Collins et al. 2018) and reduced introspection (Williams 2010) have led to an emphasis on psychoeducation and the use of behavioural techniques in autism adapted Cognitive Behaviour Therapy (CBT) (National Institute for Care and Excellence [NICE] 2012, 2013). These adapted CBT protocols have been shown to be effective in the general treatment of anxiety co-occurring with autism (e.g., Weston et al. 2016).

CBT adaptations for social anxiety in the context of autism (Spain et al. 2018) often involve teaching of adaptive social skills in a structured way using concrete and systematic prompts and incorporate elements from behavioural interventions (Heimberg 2002; Sukhodolsky et al. 2013; Sze and Wood 2008). More naturalistic forms of behavioural interventions aimed to increase social communication skills have also been found to reduce symptoms of anxiety indirectly in autistic children without explicitly targeting anxiety as a treatment goal (Lei et al. 2017).

While social skills training combined with exposure can be effective in reducing social anxiety related distress and avoidance (Beidel et al. 2014), evidence from clinical trials of SAD with neurotypical adults indicate that specific cognitive therapy for social phobia has a greater treatment effect than exposure and applied relaxation (Clark et al. 2006). Including social skills training in social anxiety treatments without the counterbalance of appraising negative social performance beliefs may carry the risk of confirming individuals’ beliefs that they are deficient in respect of social interaction skills, further exacerbating anxiety reactions and related behaviours. Furthermore, the role of prior aversive social experiences such as peer victimisation and bullying may be highly pertinent in understanding the development of negative social evaluation concerns in autism.

## Limitations and Future Directions

This study has a number of limitations regarding sample and measurement issues. Firstly, regarding the sample, there are some differences between the neurotypical and autistic students in terms of demographics and context. The current study also lacked socioeconomic status information, Participants were predominantly white, and generalisability of results to a more ethnically and socioeconomically diverse group of participants need to be explored in future studies.

Although there were gender differences across the neurotypical (predominantly female) and autism (predominantly male) samples, the heightened prevalence rate of SAD in autism may be more comparable to the rates found in neurotypical female adolescents (Pickering et al. 2019), who are more likely to report greater levels of FNE compared to their neurotypical male counterparts (La Greca and Lopez 1998; Storch et al. 2003). Therefore, the consistent FNE factor identified in the current study may suggest that this psychological construct underlying social anxiety development in autistic adolescents is more in line with that reported by neurotypical females. Future studies using more evenly distributed samples by gender may further explore the effect of diagnosis by gender interaction on the development of social anxiety in both groups.

In terms of contextual factors surrounding the time of completing the social anxiety questionnaires across the two samples, both student groups answered the questions when they encountered an unfamiliar social situation (either going to university or attending a residential summer camp and living away from home) which can be seen as an acute stressor that exacerbated the manifestation of underlying levels of trait social anxiety experienced by the students. Nonetheless, there may be broader external contextual differences between the two samples that may have affected students’ levels of state anxiety experienced in response to their immediate social environment. Future studies can further examine potential differences in the psychological constructs underlying state versus trait social anxiety in response to acute stressors in both autistic and neurotypical adolescents.

Secondly, regarding issues around measurement, although attention was paid to only include items relating to social communication difficulties from both measures of autism (AQ-28 and SRS-S) across the neurotypical and autistic student sample, it was not a perfectly direct comparison due to each group only completing one of the two measure. Future studies should seek to employ the same measures of autistic traits across both neurotypical and ASD samples to directly compare and contrast construct overlap between autism and social anxiety.

The current study also only used self-report measures for both student groups. Given that students were either seeking to transition to higher education or were first-year undergraduate students, both student groups were perceived to be competent in completing self-report measures independently. In addition, the results from bivariate correlations between measures of autistic traits and social anxiety showed a stronger magnitude in comparison to with other measures (i.e., SDQ/WEMWBS), further suggesting that there is a degree of true-comorbidity in these measures of social difficulties beyond that of shared method variance as a result of self-report measures. However, intellectual abilities do not necessarily translate into emotional literacy equally across the two student groups. There may be greater individual differences in the level of insight into one’s own social and emotional difficulties amongst autistic students compared to their neurotypical peers.

In a recent study that examined inter-rater reliability and measurement invariance using the SAS-A in both autistic adolescents and their caregivers, Schiltz et al. (2019) found that adolescents reported less severe and more infrequent symptoms of social anxiety compared to caregivers, and inter-rater differences were found at the item level as well as at the factor level when comparing differences in FNE and SAD subscale scores. Although the authors did not directly examine whether individual differences in introspection and emotional literacy contributed towards reporter bias observed, they highlighted the importance of gathering symptom ratings from multiple informants to inform clinical practice when working with autistic adolescents. Therefore, a future direction is to examine to what extent current patterns of construct overlap may be applicable to non-self-report ratings for both autistic and neurotypical students. Understanding potential influence of reporter bias is also important for including individuals with intellectual disabilities who may be unable to independently complete self-report measures, regardless of autism diagnosis. Furthermore, given that the current study included a sample of autistic participants without intellectual disability or complex co-occurring mental and physical health conditions, the generalisability of the present findings to a more heterogeneous sample of autistic individuals across the autism spectrum remains to be explored. Future studies should seek to examine whether the construct of FNE and degree of shared and unique constructs underlying social anxiety and autistic traits may be generalisable across the autism spectrum and cognitive abilities.

## Conclusion

The current study investigated possible construct overlap between measures of autistic traits, social anxiety and broader internalising symptoms and wellbeing in autistic and neurotypical adolescents. Fear of Negative Evaluation was identified as a stable construct underlying social anxiety across both samples which was distinct from the wider social communication difficulties associated with autistic traits. These findings imply that treatments for social anxiety co-occurring with autism should aim to address negative cognitions about the self and the reactions of others in social situations. Further understanding of social anxiety co-occurring with autism in young people should aim to take account of the mechanisms by which such negative cognitions develop, including a history of aversive social experiences.

## Appendix 1

### Results

#### Factor Analysis of Autistic Traits in Neurotypical Sample

An initial analysis was run to obtain eigenvalues for each factor in the data identified from the exploratory factor analysis. The Kaiser–Meyer–Olkin measure verified the sampling adequacy for the analysis, KMO = .80, which is above the acceptable limit of 0.5 (Kaiser and Rice 1974). Seven factors had eigenvalues over Kaiser’s criterion of one and in combination explained 56.31% of the variance. The scree plot also showed an inflexion after seven factors, justifying the retention of a seven-factor model. Five items loaded onto Factor one (Social difficulties), which explained 19.66% of the variance, four items (e.g., I find social situations easy; I find it hard to make new friends; New situations make me anxious) loaded uniquely and saliently (i.e., above .4 and loaded on no other factor > .4). One item (i.e., I enjoy social occasions) loaded both onto Factor 1 (0.54) and 7 (− .46). The second factor (Imagination) explained an additional 9.36% of the variance, and had four items (e.g., I find making up stories easy; If I try to imagine something, I find it very easy to create a picture in my mind) which loaded uniquely and saliently. Factor three (Fascination with numbers/patterns) explained another 7.79% of variance, and had five items (e.g., I notice patterns in things all the time; I am fascinated by dates) which loaded uniquely and saliently. Factor four (Task switching and attention) accounted for an additional 6.25% of the variance, and had three items (e.g., I find it easy to do more than one thing at a time; If there is an interruption, I can switch back to what I was doing very quickly) which loaded uniquely and saliently. Factor five (Preference for routine) accounted for 4.92% of the variance, and had three items (e.g., I prefer to do things the same way over and over again; It does not upset me if my daily routine is disturbed) with unique and salient factor loadings. Factor six (Theory of mind) accounted for 4.44% of the variance, and had three items (e.g., I find it difficult to work out people’s intentions; I find it difficult to work out what someone is thinking or feeling just by looking at their face) with salient and unique loadings. Finally, factor seven (Social motivation) accounted for 3.9% of the variance, and included three items, two items (e.g., I prefer to do things with others rather than on my own; I find myself drawn more strongly to people than to things) showed unique and salient factor loadings.

#### Factor Analysis of Autism Symptom Severity in Autism Sample

An initial analysis was run to obtain eigenvalues for each factor in the data. The Kaiser–Meyer–Olkin measure verified the sampling adequacy for the analysis, KMO = .81, which is above the acceptable limit of 0.5 (Kaiser and Rice 1974). Three factors had eigenvalues over Kaiser’s criterion of one and in combination explained 53.63% of the variance. The scree plot also showed an inflexion after three factors, justifying the retention of a three-factor model. Five items loaded onto Factor 1, which explained 34.13% of the variance, four items (e.g., I have difficulty relating to peers; I have difficulty making friends, even when trying my best) loaded uniquely and saliently (i.e., above .4 and loaded on no other factor > .4). One item (I have trouble keeping up with the flow of a normal conversation) loaded significantly both onto factor one and two. Two items loaded onto Factor 2, which explained 10.34% of the variance, with one item (I would rather be alone than with others) having loaded uniquely and significantly. Five items loaded significantly and uniquely onto Factor 3, which explained 9.16% of the variance, including (i.e., I have more difficulty than other do with changes in routine; I avoid eye contact with other people).

## Appendix 2

### Results—Relationship Between Autistic Traits and Social Anxiety

#### Neurotypical Sample

We conducted independent samples t-test to examine differences in autistic traits between the 174 neurotypical participants with elevated levels of social anxiety symptoms (above cut-off score on SAS-A), and the 93 participants who did not meet clinical cut-off. We found that participants who had higher levels of self-reported social anxiety had higher total level of autistic traits (M = 65.71; SD = 9.75) compared to those who had lower levels of self-reported social anxiety (M = 57.12; SD = 7.49; *t*(232.46) =  − 8.02, *p* < .001, 95% CI [− 10.71, − 6.48]). When the items related to social difficulties (5 items), theory of mind (3 items), and social preference (2 items) were removed from the total score, participants with higher social anxiety still showed greater levels of autistic traits (High: M = 42.23, SD = 6.14; Low: M = 37.96, SD = 5.47; *t*(265) = -5.63, *p* < .001, 95% CI [− 5.77, − 2.78]). Similar to White et al. (2012), results suggest that those who are high in social anxiety have greater autistic traits that cannot be simply accounted for by construct overlap in social anxiety and social communication difficulties between AQ-28 and SAS-A.

#### Autism Sample

We conducted independent samples t-test to examine differences in self-reported autism symptom severity between the 109 autistic participants with elevated levels of social anxiety (SAS-A total score > 50), and the 36 students who did not meet clinical cut-off. We found that participants who had higher levels of self-reported social anxiety (M = 19.36, SD = 5.56) had higher total level of autism symptoms (M = 14.69, SD = 5.77; *t*(143) =  − 4.32, *p* < .001, 95% CI [− 6.80, − .2.53]). When assessing the three factors derived from the SRS-S factor analysis, students who had greater social anxiety also reported greater social difficulties on the SRS-S (factor 1) (*t*(143) =  − 4.28, *p* < .001, 95% CI [− 3.51, − 1.29]), and greater sensory and other difficulties (factor 3) (*t*(143) =  − 3.64, *p* < .001, 95% CI [− 3.03, − .90]), suggesting that autistic students with greater social anxiety experience great severity across a wider range autism related difficulties, beyond that of social difficulties. No differences in social motivation (factor 2) (*t*(143) =  − 1.42, *p* = .158, 95% CI [− 0.54, 0.09]) were found.

## Appendix 3

### Results—Exploratory Factor Analysis of Warwick Edinburgh Mental Wellbeing Scale

#### Autism Sample

We conducted an exploratory factor analysis using oblique rotation (direct oblimin) on the 14 items of the WEMWBS. The Kaiser–Meyer–Olkin measure verified the sampling adequacy for the analysis, KMO = .80, which is above the acceptable limit of 0.5 (Kaiser and Rice 1974). An initial analysis was run to obtain eigenvalues for each factor in the data. Four factors had eigenvalues over Kaiser’s criterion of one and in combination explained 61.58% of the variance. The scree plot also showed an inflexion after four factors, justifying the retention of a four-factor model. Five items loaded onto Factor 1, which explained 36.56% of the variance, all items (e.g., I’ve been feeling close to other people; I’ve been feeling interested in other people) loaded uniquely and saliently (i.e., above .4 and loaded on no other factor > .4). The second factor explained 10.04% of the variance, and had two items (i.e., I’ve been dealing with problems well; I’ve been able to make up my own mind about things) which loaded uniquely and saliently. Factor three explained 7.77% of the variance, and had three items (i.e., I’ve had energy to spare; I’ve been feeling relaxed; I’ve been thinking clearly) which loaded uniquely and saliently. Factor four explained 7.21% of the variance, and had three items (i.e., I’ve been interested in new things; I’ve been feeling confident; I’ve been feeling optimistic about the future) which loaded uniquely and saliently. Based on these findings, the four factors seemed to correspond to: (1) social wellbeing and relationships; (2) decision making/problem solving; (3) mental wellbeing; (4) self-esteem and future.

## Footnotes

1 In a study that examined preference for use of terminology when referring to autism amongst professionals, self-advocates, and other stakeholders, Kenny et al. (2016) found that members of the autism community and autistic individuals preferred to use identity-first language, as opposed to person-first language. This paper will use identity-first language, in line with preference shown by autistic individuals.
